# Parameters and Measurement Techniques of Reconfigurable Intelligent Surfaces

**DOI:** 10.3390/mi13111841

**Published:** 2022-10-27

**Authors:** Biswarup Rana, Sung-Sil Cho, Ic-Pyo Hong

**Affiliations:** 1 Smart Natural Space Research Centre, Kongju National University, Cheonan 31080, Korea; 2 Department of Smart Information Technology Engineering, Kongju National University, Cheonan 31080, Korea

**Keywords:** reconfigurable intelligent surface, unit cell, polarization independent, measurement

## Abstract

Reconfigurable intelligent surface (RIS)-aided wireless communications systems are one the promising wireless communication system where the wave can be guided by the RIS. It is envisioned that beyond-5G/6G communication will have a low-cost, high spectral efficiency, high energy efficiency, and smart wireless environment. In this paper, initially, different measurement techniques of the RIS have been discussed, which are available in the literature. Then, a new type of RIS has been proposed. Finally, a different parameter measurement technique for our proposed RIS has been presented. A low-cost FR4 substrate with a height of 1.6 mm was considered to design the RIS in the sub-6 GHz frequency band. Another important thing is that our proposed IRS is a single-layer substrate backed by a copper plate. The area of each unit cell was 42 mm × 42 mm. The RIS was designed to operate at the central frequency of the 3.5 GHz frequency band. The novelty of the proposed RIS is that it is a polarization-independent structure. Thus, polarization-related losses can be overcome using this structure. A 10×10-unit cell array was designed to check the radiation performance. The magnitude of the reflection coefficients was measured in our laboratory for the proposed configuration.

## 1. Introduction

Wireless communication engineers have imagined the seamless connectivity of current 5G and future 6G cellular communications for everyone and everything [1,2]. Currently, the 5G communications system uses massive multiple-input multiple-output (MIMO), where 64 or more antenna elements are being used to send the beam in the desired direction [3,4]. Using this technique, efficiency can be improved significantly. However, 6G will have some applications such as augmented reality [5], virtual reality [6], mixed reality, brain–computer interface [7], autonomous cars [8], connected robotics, connected unmanned aerial vehicles [9], connected satellites, etc. Current 5G communications cannot fully support those applications. It is envisioned that 6G communications will have ultra-reliability ultra-low latency (end-to-end latency 0.1 ms) [10], higher data rate (peak data rate 1000 Gb/s) [10], energy-efficient and spectral-efficient communication systems, etc. To meet the demands of such applications and such specifications, it is necessary to adopt a new type of communications system where the waves can be guided.

It is envisioned that beyond-5G/6G communications will be guided communications where the wave will be guided by the reconfigurable intelligent surfaces (RIS) [11,12,13]. The RISs generally consist of a metasurface, which is of subwavelength planar or quasi-planar unit cells, and the 2D equivalent of metamaterial [14,15]. The properties of the metamaterials and metasurface are not found in nature, it is an engineered structure. The unique properties of the metasurface or metamaterial can be used to achieve different applications such as cloaking illusions to holograms, real-time reconfiguration of wireless environments, etc. [16,17,18,19,20].

In recent years, there has been significant growth in research activity in this area [21,22,23,24]. RIS is a reflector to assist the existing communication by reflecting the incoming signal in the desired direction. It is possible to enhance the energy and spectral efficiency significantly using RIS-aided communications. The secrecy rate of a channel can be improved using RIS. Cogitative radio and non-orthogonal multiple access are other applications where RIS is very useful. RIS consist of a large number of unit cells, and each unit cell can tune the phase or magnitude of the reflected wave. This RIS can be placed on the wall of any building, and these are cost-effective and low-power consumption devices. The signal-to-noise ratio can be improved significantly to the desired location using RIS while suppressing co-channel interference. Hence, significantly improved communication performance can be obtained using RIS without additional antennas.

Recently, there have been some publications on the hardware aspect of the RIS for beyond 5G/6G applications, and several papers have discussed measurement techniques of RIS performances. In [25], the authors used varactor diodes to design RIS in the sub-6 GHz frequency band. Overall, 2430-unit cells were considered to design the RIS. To measure the performance of the RIS, the authors placed the transmitting horn antenna 30 m from the RIS. The authors used software define radio (SDR) to send a video signal using QPSK modulations. In [26], the authors proposed varactor diodes based on an RIS working at 5.8 GHz. The proposed RIS had a total of 1100 controllable unit cells. The channel reciprocity and reflection coefficient was measured using a vector network analyzer. In that experiment, the horn antenna was placed very close to RIS. The authors also tested the RIS in a long-distance measurement scenario with a distance of 50 m and 500 m. A spectrum analyzer was used in the outdoor environment to measure the spectrum of the received signal. In [27], the authors proposed a 256 elements new type of high-gain yet low-cost RIS that combines the functions of phase shift and radiation on an electromagnetic surface. The 2-bit operation was conceived using PIN diodes for beamforming operations. In [28], the authors proposed a new type of PIN diode-based RIS working at mmWave. The authors measured the reflection property of the unit cell using a WR-34 rectangular waveguide. The waveguide was connected to Rohde & Schwarz ZVA-40 vector network. After that, an FPGA control board was used to connect each unit cell of the RIS. There was a total of four ports in the control board, which can connect to four independent RIS. Lastly, the control board was connected to the desktop using a USB interface or LAN interface. MATLAB code or Phyton code was used to control the FPGA board and, consequently, each unit cell of the RIS. In [29], the authors proposed a new type of unit cell working at 5.4 GHz. The authors used computer vision to aid the RIS for dynamic beam tracking. A camera was used on the RIS to track the visual information of the surrounding area, and the reflected beam was able to go in the desired direction. A 20 × 20 RIS was fabricated, and the performance of the RIS was verified. The authors utilized a machine learning algorithm to calculate the vision disparity. The proposed RIS worked under near-field conditions and far-field conditions. The RIS can work as a passive array for beam-forming operations in one case, and in another case, the RIS assists the communication between the base station and user equipment. To test the performance of the RIS, two horn antenna and an FPGA board was used. In [30], the authors proposed PIN diodes based on 160 elements of RIS working at 5.8 GHz. To test the RIS’s performance, the authors initially placed the RIS in an anechoic chamber and used horn antennas. The authors also undertook a field test of the proposed RIS. In [31], the authors proposed a high-accuracy 2-bit RIS. To check the performance of the RIS, an FPGA board, a module for demultiplexing, a transmitting antenna, and two receiving antennae were used. Each element of the RIS could be controlled by FPGA through DuPont l. To overcome path loss and shadowing effects, the authors proposed RIS-aided communications by using a compress sensing-based algorithm [32]. The steering capabilities of the RIS were verified by measuring the radiation pattern of the RIS. Software defined radio NI USRP 2944, pair of horn antennae for transmitting and receiving the signal were used to measure the parameters of the RIS. Not only reflective types of RIS but also transmissive types of RISs are also going to play a very significant role in beyond-5G/6G communications. In [33], a new type of 2-bit transmissive RIS was proposed. To measure the properties of the RIS, both the receiver and transmitter used an SDR platform. There were transmitter hosts, FPGA, the analog to digital converter, mmWave upconverter, and a horn antenna in the transmitter section. In the receiver section, there were receiver hosts, the FPGA, digital-to-analog converter, mmWave down converter, and a horn antenna. In [34], the authors proposed a 3-bit reconfigurable digital metasurface. To experimentally characterize, the authors fabricated a 6×6 array of the unit cell containing a total of 144 varactor diodes. The receiving and transmitting horn antennas were connected to the two ports of the VNA to measure the complex forward transmission coefficient. To provide eight stable DC voltages to the metasurface, a power supply was connected. In [35], the authors proposed a multi-bit RIS capable of beam steering at the sub-6 GHz frequency band. To measure the performance of the RIS, a USRP-based communication link was used in different scenarios, such as indoor mixed LoS/NLoS, corridor junctions, and multiple floors. In [36], metamaterial-based RIS was proposed based on the 3D use of 3D graphene meta-atoms. The reconfigurability of the RIS can be achieved by setting the discrete state (ON/OFF) of the graphene. A new type of RIS surface with sensing capabilities was proposed in [37]. A small portion of the signal was coupled, and it was used to sense the angle of arrival of the incoming signal. In [38], an RF switch-based RIS was proposed, which enables a passive type of 3D beam, forming without the use of any active RF components. To measure the performance of the proposed RIS, the authors mounted the RIS on a controller remotely from a master PC. Two SDR devices were attached to a horn antenna with a gain of 13.5 dBi. Vanadium dioxide is a promising material for RIS. In [39], authors proposed a vanadium dioxide-based RIS for 5G applications. Metal-to-insulator property of the vanadium dioxide was used for the reconfigurable operations. In [40], the authors reported a prototype of a RIS engineered to operate in the frequency range of 5.15–5.75 GHz.

In this paper, a new type of passive unit cells has been proposed, which is polarization independent by nature. The polarization independence feature allows the acceptance of any type of incoming polarization without any polarization-related losses. The unit cells can be arranged in a particular manner to achieve passive-type RIS. The reconfigurability of the proposed RIS can be achieved by rotating the RIS using a mechanical motor. We have designed this type of RIS mainly to measure the different parameters of the RIS and set up a measurement environment in our laboratory for any kind of RIS measurement. We have checked the different parameters of our proposed RIS. 

## 2. Design of Unit-Cells

**Figure 1:**
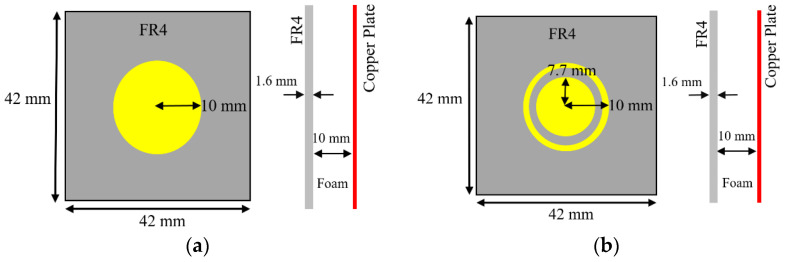
Top view and side view of the unit cell (**a**) circular patch (**b**) circular patch with a ring.

**Figure 2:**
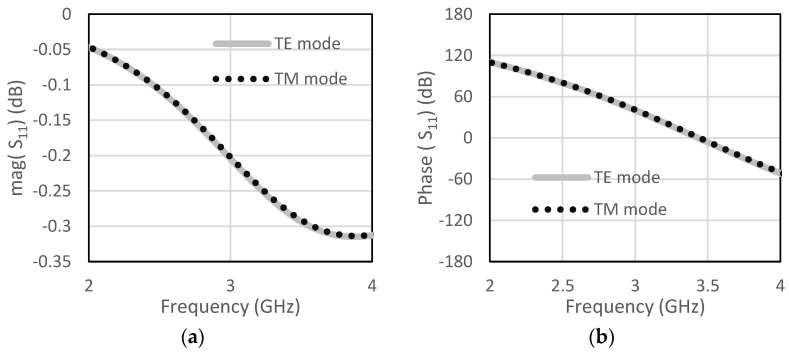
Circular type of patch (**a**) magnitude of S_11_ (**b**) phase of S_11_.

**Figure 3:**
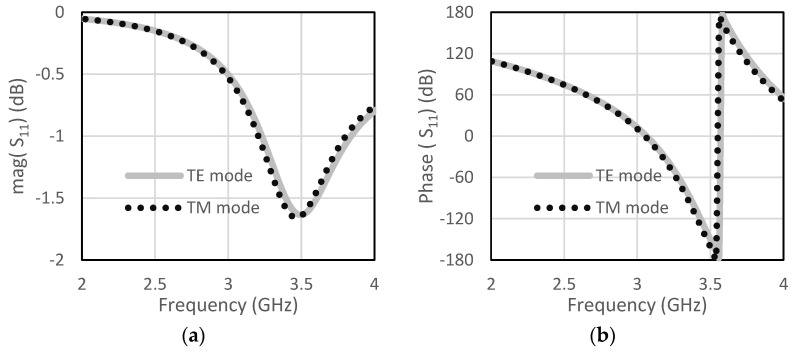
Circular patch with a ring (**a**) magnitude of S_11_ (**b**) phase of S_11_.

**Figure 4:**
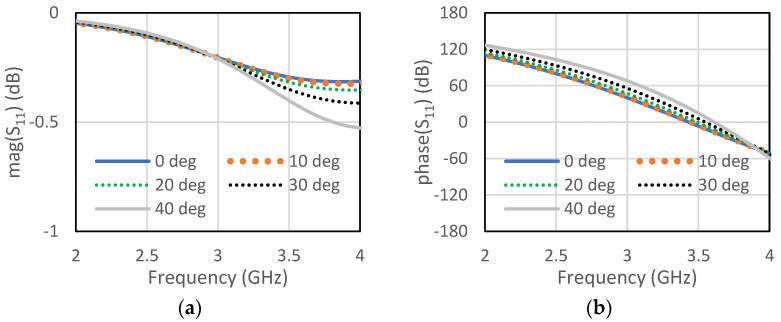
Circular type of patch (**a**) magnitude of S_11_ for different angles of incidence (**b**) phase of S_11_ for different angles of incidence.

**Figure 5:**
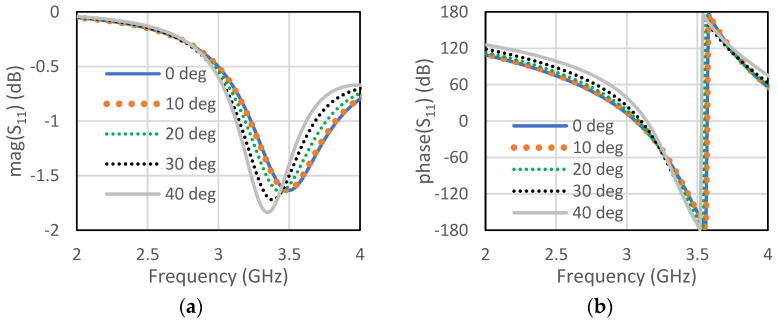
Circular patch with a ring (**a**) magnitude of S_11_ for different angles of incidence (**b**) phase of S_11_ for different angles of incidence.

**Figure 6:**
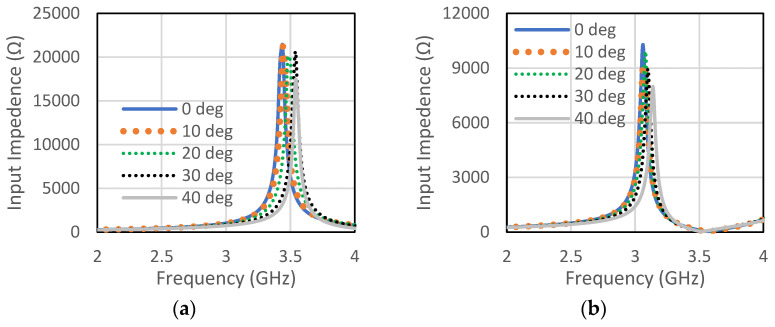
The magnitude of the input impedance of the (**a**) circular patch and (**b**) circular patch with a ring.

The unit cell for the proposed RIS was designed at the center frequency of 3.5 GHz. The size of the unit cell was 42 mm × 42 mm. A low-cost FR4 substrate with a thickness of 1.6 mm, loss tangent of 0.002, and dielectric constant of 4.4 was considered for our design. The single-element unit cell was taken as a base element for the RIS design. Initially, a circular patch with a radius of 10 mm was taken on the FR4 substrate, as shown in Figure 1a. There was a copper plate below 10 mm from the dielectric for the reflections of the incoming waves. This type of unit cell was considered for the RIS design due to its simple construction and low cost. The circular-type patch and the circular type-patch with a ring were chosen to make the structure polarization independent. Polarization-mismatch related losses can be overcome using this type of polarization-independent structure. Figure 1b shows the circular patch with a circular type of ring to achieve a similar type of magnitude of the reflection coefficient, but almost 180° shifted phase. The magnitude and phase of the S_11_ parameters for the unit cell of circular type patch are shown in Figure 2a,b, respectively, for both the TE and TM mode. The polarization-independent nature of the unit cell can be verified from their modes. It was observed from Figure 2 that the magnitude of the reflection coefficient was −0.29 dB for both TE and TM modes at 3.5 GHz. The phase of the reflection coefficient was −5° for both TE and TM modes at 3.5 GHz. Figure 3a,b show the magnitude and phase of the reflection coefficient at 3.5 GHz for both TE and TM modes. The magnitude and phase of the reflection coefficient were −1.6 dB and −152°, respectively, for both TE and TM modes. The performance of the unit cells under different angles is a very important factor. The performances of unit cells under incidence angle of 0°,10°, 20°,30°, and 40° has been presented here for both types of unit cells. Figure 4a,b show the magnitude and phase of the circular type of patch unit cell for 0°,10°, 20°,30°, and 40° angles of incidences. It can be observed from these Figures that there are no significant changes in the performances of the unit cells under the different angles of incidence. Figure 5a,b show the magnitude and phase of the circular type of patch with a ring unit cell for 0°,10°, 20°,30°, and 40° angles of incidences. In this case, the performances of the unit cell were also not changed significantly. Input impedances of the circular type of unit cell and circular type of unit cell with a ring are presented in Figure 6a,b, respectively.

## 3. Radiation Performance Test for 10×10 Unit Cells

**Figure 7:**
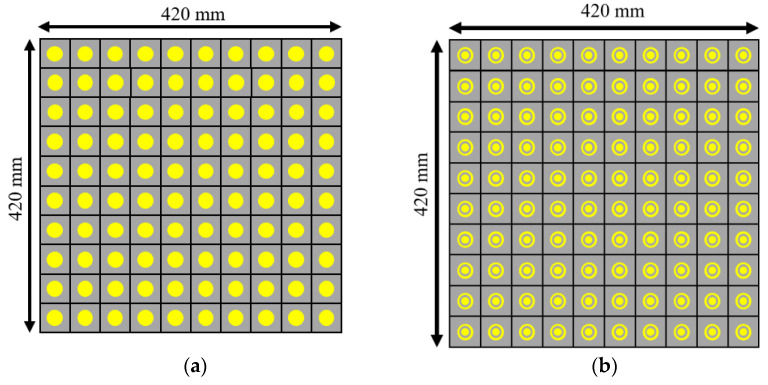
10×10-unit cells array (**a**) circular type patch (**b**) circular type patch with a ring.

**Figure 8:**
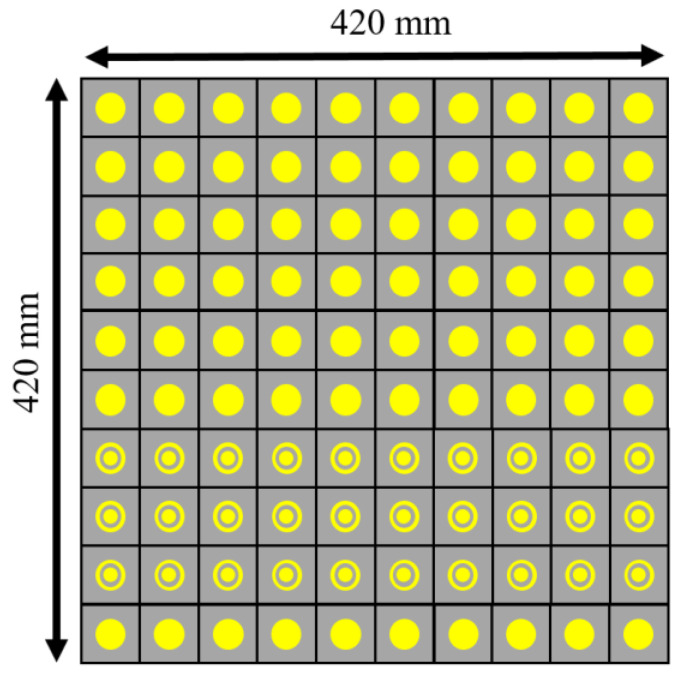
10×10-unit cell array with combined structure.

**Figure 9:**
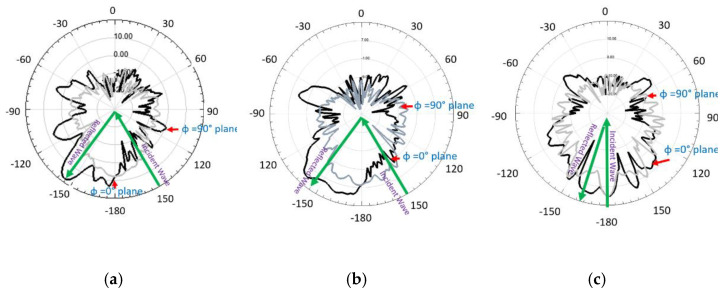
Radiation patterns (**a**) 10×10 circular type patch array (**b**) 10×10 circular type patch with a ring array (**c**) 10×10 combined array.

**Figure 10:**
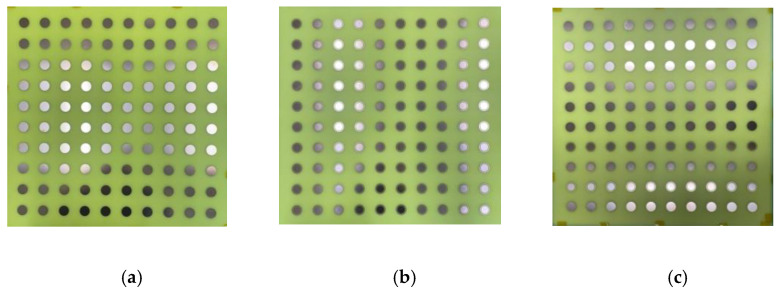
Fabricated prototypes (**a**) 10×10 circular type patch array (**b**) 10×10 circular type patch with a ring array (**c**) 10×10 combined array.

To check the radiation performance of the unit cells, we took the arrays of the 10×10-unit cells shown in Figure 7. Figure 7a shows the circular patch type 10×10-unit cells while Figure 7b and Figure 8 show the circular type of patch with a ring 10×10-unit cells and the combined type of the 10×10-unit cells array, respectively. Standard horn antennas working at 3.5 GHz were in our design. The radiation from the standard horn antenna was impinging upon the 10×10-unit cells. We studied the reflected radiation patterns for the 10×10 circular patch array, 10×10 circular path with ring array, and 10×10 combined array. It is noted that the radiation pattern performances for these types of arrays are not very important. We have still included the radiation patterns for all those arrays. The real-time communication performances using those arrays are very important to study for such types of arrays. However, in this paper, the real-time performances using communications links are not presented. To check the radiation performances of these arrays, a standard horn antenna working at 3.5 GHz was considered. For the 10×10 circular patch type array, there was a transmitting horn antenna which was placed at an angle of 30° from the array. The waves were impinging on the surface of the arrays and were reflecting from the surface. To collect the reflected signal from the array, a receiving horn antenna was placed at −30° with the array. The same procedure was followed for the 10×10 circular patch type with a ring array. However, for the combined 10×10 arrays, the transmitting horn antenna was placed at an angle of 0° to the combined 10×10 arrays while the receiving antenna was at a certain angle. Figure 9a shows the reflected radiation pattern from the 10×10 circular type of array, while Figure 9b,c show the reflected radiation patterns from the 10×10 circular type with a ring array and the combined array, respectively. The peak gain of the incident pattern was 13.2 dBi for both φ = 0° and φ = 90° planes for all three cases. The peak gains for the reflected patterns for the circular type of patch and circular type of patch with a ring were 11.2 dBi and 18.1 dBi, respectively. The proposed fabricated arrays are shown in Figure 10. Figure 10a–c show the fabricated 10×10 circular type patch type of arrays, circular type of array with a ring, and combined structure, respectively.

## 4. Measurement Technique of Our Proposed RIS

**Figure 11:**
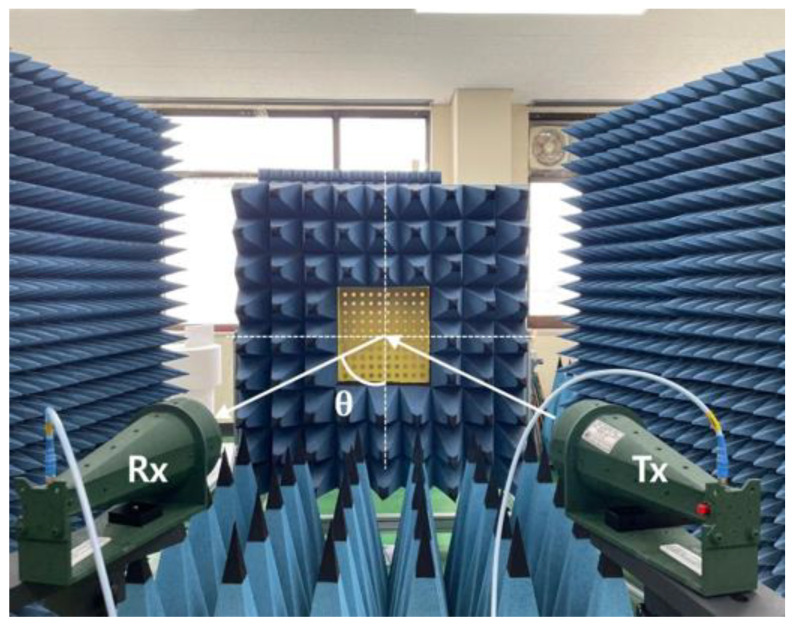
Measurement setup to measure different parameters.

**Figure 12:**
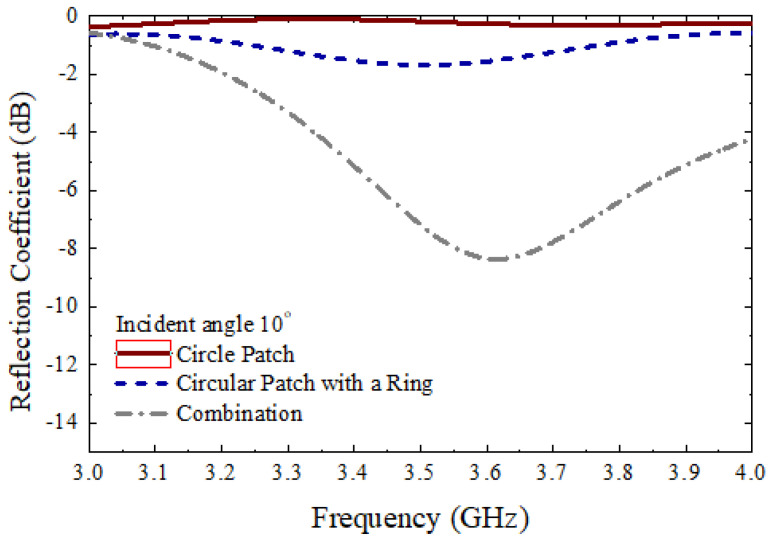
Measured reflection coefficients for three different types of arrays at an incident angle of 10°.

**Figure 13:**
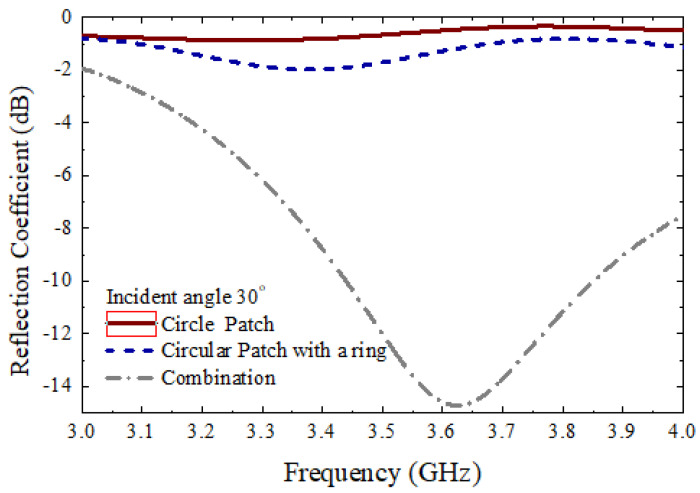
Measured reflection coefficients for three different types of arrays at an incident angle of 30°.

Figure 11 shows the measurement setup to measure the different parameters. The proposed 10×10 circular type patch array or the 10×10 circular path array with a ring, or the 10×10 combined array can be placed middle of the main absorbing material, as shown in Figure 11. There were several absorbers left side and right side of the main absorber. Additionally, there were absorbers on the ground. The purpose of adding more absorbers is to reduce the unwanted radiation from outside as well as reduce the reflection from the wall of the room. As shown in Figure 11, two standard horn antennas working at 3.5 GHz were considered to measure the different parameters of the 10×10 arrays. The antennas were connected to the vector network analyzer (VNA) to measure the reflection coefficients of the proposed arrays. To measure the reflection coefficients of three types of 10×10 arrays, initially, the transmitting horn antenna was placed at an angle of 10°, and the received powers were measured using a similar type of horn antenna at an angle of −10°. Figure 12 shows the measured reflection coefficients for three different cases at an angle of incidence of 10°, while Figure 13 shows the reflection coefficients for three cases at an angle of incidence of 30°. It is to be noted that the reflection coefficients for the combined 10×10 array were very low. This is because the beam is steered at a different angle for the combined 10×10 array. To achieve an actual good reflection coefficient for the combined 10×10 array, the receiving horn antenna needs to be placed at the steered beam position. However, in this paper, this type of measurement was not performed. Initially, a 10×10 circular type patch was designed, and the performances of the 10×10 circular type patch were tested. From the 10×10 circular type patch, it was observed that the 10×10 patch was working as a good reflector. After that, a 10×10 circular type of patch with a ring was designed and the performance of the structure was tested. It was also observed that the 10×10 circular type of patch with a ring was working as a good reflector. The unit cell of the circular-type patch with a ring has a very significant phase difference from the phase of the unit cell of the circular type. By combining these two types of unit cells, it is possible to achieve a scanned beam. A 10×10 combined array was taken so that a well-scanned beam could be obtained from the structure.

Table 1 shows the comparison of the RIS available in the literature with our proposed structure [25,26,27,28,29,30,31,32,33,34,35,36,37,38,39,40]. From the table, it can be seen that our proposed structure has some advantages. Our proposed structure has no PIN or varactor diodes. The reconfigurability of the structure can be achieved by machinal rotating the structure. Our proposed structure has only a single layer backed by the copper plate. Thus, it is very easy to fabricate, and it is cost-effective. Additionally, we do not need any complicated external controller for our structure.

## 5. Conclusions

In this paper, a polarization-independent passive type RIS has been proposed. The reconfigurability can be achieved by rotating the RIS 0°, 90°, 180°, and 270°. The unit cells were simulated, and the performance of two types of unit cells has been presented in this paper. There are some circumstances where mechanical rotating RIS is very useful compared to the PIN diode or varactor diode-based RIS. The unit cells are very simple, and it is very easy to fabricate. As the substrate to design this RIS was FR4, it is very cost-effective. We have studied the preface of the unit cells under the different angles of incidence, and it was observed that there were no significant changes in the unit cell performances under the different angles of incidence. Three types of 10×10 arrays were fabricated, and the performance of each type of array has been verified. The unit cells and arrays were designed at 3.5 GHz for sub-6 GHz frequency applications.

## Figures and Tables

**Table 1:** Comparison of different types of RIS proposed in the literature.

Ref.	Nature of Unit Cell	Year	Tunning Method	Freq.	Comments
[25]	D-type patch	2022	Varactor diode	3.5 GHz	Expensive to fabricate and need an expensive external control board
[26]	Normal patch	2021	Varactor diode	5.8 GHz	Expensive to fabricate and need an expensive external control board
[27]	Patch	2020	PIN diode	2.3 GHz, 28.5 GHz	Very complicated structure and need an external controller
[28]	Patch	2021	PIN diode	28.5 GHz	Very complicated structure and need an external controller
[29]	Joined E-shaped	2022	PIN diode	5.4 GHz	Need complicated external controller
[30]	Normal patch with a parasitic patch	2022	PIN diode	5.8 GHz	Need complicated external controller
[31]	Patch with PIN Diodes	2021	PIN diode	-	Complicated unit cell and external controller
[32]	Patch	2021	PIN diode	5.8 GHz	Multilayer unit cell
[33]	Transmissive type unit cell	2022	PIN diode	27 GHz	Complicated Structure
[34]	3-bit Unit Cell	2021	Varactor diode	5 GHz	Complicated unit cell and need an external controller
[35]	Patch	2021	PIN diode	3–4.5 GHz	Need external controller
[36]	3D graphene	2021	Graphene On/OFF	28 GHz	Difficult to fabricate the 3D structure
[37]	Patch	2021	Varactor	18.6–19.2 GHz	Need an external controller and need lots of varactor diodes
[38]	Patch	2022	RF switch	5.3 GHz	Compilated RIS board
[39]	Vanadium dioxide-based unit cell	2022	Vanadium dioxide	5 GHz, 32 GHz	Multi-layer structure
[40]	Patch separated by an annular slot	2022	Varactor diode	5.15–5.75 GHz	Need external controller
Our work	Circular patch and circular patch with a ring	-	Mechanical Rotation	3.5 GHz	Single layer unit cell backed by copper plate, easy to fabricate, low cost, no external complicated controller
